# Higher Bacterial Diversity of Gut Microbiota in Different Natural Populations of Leafhopper Vector Does Not Influence WDV Transmission

**DOI:** 10.3389/fmicb.2019.01144

**Published:** 2019-05-29

**Authors:** Hui Wang, Nan Wu, Yan Liu, Jiban Kumar Kundu, Wenwen Liu, Xifeng Wang

**Affiliations:** ^1^State Key Laboratory for Biology of Plant Diseases and Insect Pests, Institute of Plant Protection, Chinese Academy of Agricultural Sciences, Beijing, China; ^2^Division of Crop Protection and Plant Health, Crop Research Institute, Prague, Czechia

**Keywords:** European grass feeding leafhopper (*Psammotettix alienus*), 16S rDNA high-throughput sequencing, gut bacterial community, wheat dwarf virus (WDV), geographic location

## Abstract

The bacterial communities in the gut of an insect have important ecological and functional effects on the insect. However, the community composition and diversity of the gut microbiota in insects that vector plant viruses are poorly understood. As an important insect vector, *Psammotettix alienus* transmits various viruses including wheat dwarf virus (WDV). Here, we used the combination of leafhopper and WDV as model to survey the influence of gut microbiota on virus transmission characteristic of insect vector and vice versa. We have characterized 22 phyla and 249 genera of all gut bacterial communities in the leafhopper populations collected from six geographic regions in China. Community composition and diversity varied across different geographic populations. However, WDV transmission efficiencies of these six field populations were all greater than 80% with no significant difference. Interestingly, the transmission efficiency of WDV by laboratory reared insects with decreased gut bacterial diversity was similar to that of field populations. Furthermore, we found that the composition of the leafhopper gut bacteria was dynamic and could reversibly respond to WDV acquisition. Higher bacterial diversity and abundance of gut microbiota in different leafhopper populations did not influence their WDV transmission efficiency, while the acquisition of WDV changes gut microbiota by a dynamic and reversible manner. This report provides insight into the complex relationship between the gut microbiota, insect vector and virus.

## Introduction

Almost all insects harbor gut microbial communities that play important functions for their hosts (Dillon and Dillon, 2004). In general, such microbial partners can provide nutrition, contribute to host reproduction and survival (Broderick et al., 2006; Sharon et al., 2010), mediate detoxification of insect diets (Genta et al., 2006; Cejanavarro et al., 2015), or confer resistance to insecticides (Kikuchi et al., 2012; Cheng et al., 2017). In addition, some microbes can help or inhibit pathogen transmission (Douglas, 2015). *Wolbachia* inhibits replication of dengue virus in the vector mosquito (*Aedes aegypti*; Moreira et al., 2009). On the contrary, the gut microbiome of the vector sand fly (*Lutzomyia longipalpis*) is essential for survival of *Leishmania infantum*, because successive daily antibiotic treatments inhibited growth and development of the parasite into its infectious metacyclic forms (Kelly et al., 2017). In addition, the composition and diversity of insect gut microbes are influenced by the external factors, such as climate change (Sheik et al., 2011), soil attributes (da C Jesus et al., 2009; Young et al., 2018), pathogens and ingested food (Knief et al., 2010; Ben Guerrero et al., 2016).

Hemipteran insects have a needle-like sucking stylet, and some are notorious agricultural pests that cause serious economic loss not only by directly sucking the plant sap but also by transmitting plant viruses (Wang et al., 2015; Liu W. et al., 2018; Qin et al., 2018). Hemipterans that are phytophagous usually feed on nutritionally deficient xylem or phloem diets, but endosymbionts in these insects can provide essential amino acids and other nutrients (Redak et al., 2004). Most leafhoppers (*Hemiptera*: *Cicadellidae*) harbor the bacterium *Candidatus Sulcia muelleri*, which coexists with other bacteria such as *Candidatus Baumannia cicadellinicola*, *Candidatus Zinderia insecticola*, *Candidatus Nasuia* or *Hodgkinia cicadicola* (Koga et al., 2013). In addition to obligate endosymbionts, leafhoppers also host various facultative endosymbionts such as *Wolbachia*, *Rickettsia* or *Cardinium* (Zheng et al., 2017).

The European grass feeding leafhopper (*Psammotettix alienus*) can lead to great yield losses by transmitting viruses such as Russian mosaic virus (Vacke, 1961), wheat yellow striate virus (Liu Y. et al., 2018) and wheat dwarf virus (WDV) in a persistent circulative manner (Wang et al., 2014). Periodic outbreaks of wheat dwarf disease outbreaks have caused economic losses in European (Benkovics et al., 2010), African (Najar et al., 2000) and Asian countries (Zhang et al., 2010), with the incidence of wheat dwarf disease in Swedish wheat fields reaching 90% in severe cases (Lindblad and Waern, 2002). In China, the first WDV disease outbreak reached up to 80% incidence, and yield was reduced by 50–80% in Hancheng, Shaanxi Province in 2007 (Wang et al., 2008). High population numbers and expanding distribution of *P. alienus* are important factors contributing to these epidemics.

Similar to viruses transmitted in a persistent circulative manner (Mar et al., 2014), WDV invades the midgut via receptor-mediated endocytosis and spreads into the salivary glands through the hemolymph, but it also rapidly moves to the hemocoel through the filter chamber (Wang et al., 2014). Interestingly, some endosymbionts are involved in the spread of viruses within the insect vectors (Kliot and Ghanim, 2013). For example, the GroEL protein of the endosymbiont *Buchnera* is crucial for determining the persistent nature of potato leafroll virus in *Myzus persicae* (Hogenhout et al., 2000). Similarly, the GroEL protein produced by *Arsenophonus* in the Asia II genetic group of *Bemisia tabaci* can interact with the viral coat protein encoded by cotton leaf curl virus (CLCuV) and is involved in virus transmission (Rana et al., 2012). Also, in *B. tabaci*, *Hamiltonella* produces a GroEL protein that is involved in transmission of tomato yellow leaf curl virus (TYLCV; Gottlieb et al., 2010; Ghanim, 2014). Recently, a symbiotic bacterium, *Sulcia*, in a leafhopper was found to directly mediate transovarial transmission of rice dwarf virus (Jia et al., 2017).

Although microbes in insect vectors might be involved in virus transmission, little is known about the change/function of insect vector gut microbiota in virus transmission. The present study wants to comprehensively characterize the bacterial communities in the gut of *P. alienus* from six locations in China by high-throughput sequencing. We also focused on the gut bacterial community changes during WDV acquisition (Supplementary Figure S1). In general, the gut bacterial communities of the field-collected leafhoppers represented 22 phyla and 249 genera, meanwhile the difference of gut bacterial community composition and diversity of the six field populations do not influence the virus transmission. Interestingly, the WDV transmission efficiency of laboratory reared leafhoppers with decreased gut bacterial diversity is similar to field populations. Moreover, composition of the leafhopper gut bacterial communities during the acquisition period was dynamic and reversible over time.

## Materials and Methods

### Leafhopper Collection

In China, wheat dwarf disease occurs frequently in Tianshui (Gansu Province), Hancheng (Shaanxi Province), Taiyuan (Shanxi Province) and Linfen (Shanxi Province), but occasionally in Baoding (Hebei Province), Shijiazhuang (Hebei Province) and Tianjin (Zhang et al., 2017). Based on the epidemic pattern of the disease, vector leafhoppers (*P. alienus*) were collected from six regions in China (Tianshui, Hancheng, Linfen, Taiyuan, Baoding and Tianjin) during April 2017 (Table 1 and Supplementary Figure S2), and taken immediately on healthy wheat seedlings in different tubes to the laboratory for transmission assay and 16S rDNA sequencing.

**Table 1:** Sampling locations and dates in 2017 for *Psammotettix alienus* and number of reads obtained from high-throughput Illumina sequencing of gut microbiome.

Location (Date)	Latitude	Longitude	Number of reads	
			Raw	Clean
Tianshui, Gansu; TS (10 April)	N34°34′5.33″	E105°39′31.62″	75,184	66,979
			75,954	68,176
			75,068	67,556
Hancheng, Shaanxi; HC (12 April)	N35°29′14.28″	E110°26′10.48″	75,760	68,465
			72,322	65,084
			75,482	68,260
Linfen, Shanxi; LF (14 April)	N36°04′56.50″	E111°36′41.05″	76,373	69,173
			76,017	68,652
			76,064	68,415
Taiyuan, Shanxi; TY (15 April)	N37°46′34.09″	E112°34′30.20″	76,212	69,030
			74,751	67,630
			75,890	68,352
Baoding, Hebei; BD (19 April)	N38°46′14.04″	E114°58′36.86″	74,702	66,993
			75,019	67,366
			75,216	67,707
Tianjin; TJ (20 April)	N39°04′38.23″	E117°03′44.64″	75,588	67,488
			76,184	68,717
			75,465	67,530


Similarly, we selected more than 200 various instars laboratory-maintained leafhoppers (collected from Linfen in 2011) and then placed them on WDV-infected wheat to feed. After an acquisition access period (AAP) of 5, 10, and 20 d, 15 adult leafhoppers as one replicate (three repetitions for each time) were collected, dissected and extracted total DNA from guts for sequencing, respectively. Nonviruliferous adult leafhoppers (1 to 3 days old) were fed on healthy wheat as controls. WDV transmission efficiency was measured as above.

### DNA Extraction From Excised Guts

Adults of *P. alienus* were first surface-sterilized with 70% ethanol for 1 min, then washed three times for 1 min each with ddH_2_O. Fifteen guts from the same population were collected in one tube as one replicate (three repetitions for every field population). DNA was extracted from all samples using a Wizard genomic DNA isolation kit (Promega, Madison, WI, United States) according to the manufacturer’s instruction.

### PCR Amplification of 16S rDNA

Genomic DNA of the gut microbiome of leafhoppers was amplified by primers V3-V4F (5′-ACTCCTACGGGAGGCA GCA-3′) and V3-V4R (5′-GGACTACHVGGGTWTCT AAT-3′) specific for the 16S rDNA hypervariable V3-V4 region. The PCR mix contained 1.5 μl PrimerF, 1.5 μl PrimerR, 0.5 μl Q5 High-Fidelity DNA Polymerase (TaKaRa, Dalian, China), 10 μl High GC Enhancer (TaKaRa), 10 μl 5 × PCR Buffer (TaKaRa), 1 μl dNTP (TaKaRa) and 40ng DNA and ddH_2_O. The thermal cycling conditions for the indexing PCR consisted of an initial denaturation at 95°C for 5 min, followed by 30 cycles of 95°C for 1 min, 55°C for 1 min and 72°C for 1 min. The PCR products were purified, quantified and amplified again as template by Solexa PCR with an initial denaturation of 98°C for 30 s, followed by 40 cycles of 98°C for 10 s, 65°C for 30 s and 65°C for 30 s. Solexa PCR products were purified and quantified for high-throughput sequencing (Illumina) by Biomarker Technologies (Beijing, China).

### Sequence Data Analyses

After sequencing, sequences were trimmed and assembled by Flash (version 1.2.7, http://ccb.jhu.edu/software/FLASH/; Magoc and Salzberg, 2011), and reads that could not be assembled were discarded. Chimeras were identified and removed using Uchime (Mothur; version 1.31.2, http://www.mothur.org/; Edgar et al., 2011). The cleaned Fastq data were aligned into operational taxonomic units (OTUs) by UCLUST (Edgar, 2010; QIIME; Caporaso et al., 2010) based on a similarity of 97%. OTUs were assigned taxons using the RDP CLASSIFIER (version 2.2, http://sourceforge.net/projects/rdp-CLASSIFIER) against the Silva database^1^. Relative OTU abundances were summarized across taxonomic levels from phylum to genus. The raw data were available under SRA accession number PRJNA495407.

### Diversity Analyses

ACE and Chao 1 indices for alpha diversity, which reflects the diversity and richness of individual samples, were plotted using the Mother package (QIIME)^2^. Beta diversity was determined to evaluate the degree of similarity of gut bacterial communities from different samples using QIIME. Principal coordinate analysis (PCoA; Sakaki et al., 1994), heat maps, dendrograms based on unweighted pair-group method with arithmetic mean (UPGMA; Kim et al., 2017) and nonmetric multidimensional scaling (NMDS; Looft et al., 2012) were used to analyze the beta diversity.

### Analysis of Significant Differences in Relative Abundance of Gut Microbes

To discover biomarkers that differed significantly among populations, we used a linear discriminant analysis (LDA) of effect size to determine OTUs that discriminate among the leafhopper populations with an LDA score is more than 4.0. Colors were used to indicate the different populations of the phylogenetic component contributing to group uniqueness. A cladogram was also constructed to show the LDA results. Levels of the cladogram represented, from the inner to outer rings, phylum, class, order, family, and genus. Color codes indicated the condition, and letters indicated the taxa that contribute to the uniqueness of the corresponding populations at an LDA score greater than 4.0.

### Function Analysis

To predict putative KEGG functions for the gut microbiome of leafhoppers from different geographic locations, we compared the predicted relative abundances of KEGG orthologs based on evenly rarefied 16S rDNA gene amplicon sequences using PICRUST version 1.0.0 (Parks et al., 2014). Fisher tests were used to compare the KEGG function predictions for the six populations.

### WDV Transmission Assay

WDV transmission efficiency of the different leafhopper populations was determined using 50 leafhoppers that were allowed to feed on WDV-infected wheat for 3 days. They were then transferred to wheat seedlings (1 insect/plant) for a 72-hour inoculation access period, and the seedlings were then grown in a greenhouse. At 21 days post-inoculation, wheat plants were analyzed for virus symptoms and tested using specific primers for WDV infection by PCR with primer pairs 5′-ATGGTGACCAACAAGGAC-3′ and 5′-TAACACGCGTGCGTATAGGC-3′ (Zhang et al., 2010). The experiments were performed three times.

In addition, we compared the WDV transmission efficiency of the laboratory-maintained leafhoppers (from Linfen) and field population.

## Results

### Sequencing Quality and Operational Taxonomic Unit (OTU) Analysis

**FIGURE 1:**
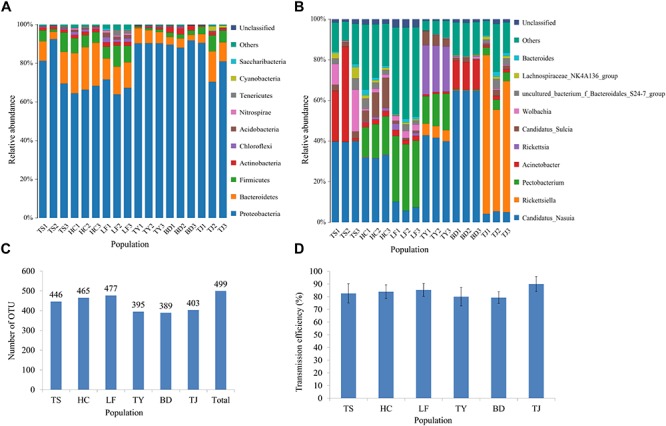
Analysis of gut microbiota and transmission efficiency of wheat dwarf virus for the field populations of leafhoppers from six locations in China. Taxonomic composition at the phylum level **(A)** and genus level **(B)**; The number of OTUs **(C)**; WDV transmission efficiency **(D)**. Only the top 10 most abundant taxa are shown for each. Results are shown for three bulked samples (*N* = 15 adults/sample) for each geographic population. The bacterial community composition varied among the locations. TS: Tianshui; HC: Hancheng; LF: Linfen; TY: Tianjin; BD: Baoding; TJ: Tianjin.

To study the composition and diversity of the gut bacterial communities in the leafhopper field population, three DNA pools of leafhoppers were extracted from each of the six geographic locations (Table 1), and then subjected to Illumina sequencing of 16S rDNA. The all samples generated 1,436,533 raw reads in total. Following demultiplexing, quality filtering and chimera removal, 1,221,573 clean tags were retained for all samples, and every sample had average 67,865 high-quality sequences. In all, 499 distinct OTUs were recognized with 97% similarity cutoff (Figure 1C).

The OTUs were assigned to taxonomic groups using the BLAST algorithm in a search against the SILVA ribosomal RNA gene database ^3^. The identified sequences were distributed across 22 assigned bacterial phyla. Members of *Proteobacteria, Bacteroidetes, Firmicutes* and *Actinobacteria* had a cumulative relative abundance of more than 80% (Figure 1A). The remaining phylum all had a very low abundance. Overall, 249 genera were represented (Figure 1B and Supplementary Table S1). At the genus level, *Acinetobacter*, *Candidatus_Nasuia* and *Wolbachia* were the three main genera in the Tianshui population, but *Pectobacterium, Candidatus_Nasuia* and *Candidatus_Sulcia* were three most abundant genera in the Hancheng population (Figure 1B). Similarly, the dominant genus in the Linfen population was *Pectobacterium*, followed in order by *Candidatus_Nasuia, Wolbachia, Rickettsia* and *Acinetobacter* (Figure 1B). In addition, two genera *Ruminobacter* and *Prevotellaceae_UCG-004* predominated in the Linfen population. *Candidatus_Nasuia, Pectobacterium* and *Rickettsia* were the three most abundant in order in the Taiyuan population. The relative abundance of *Candidatus_Nasuia* surpassed 60% in the Baoding population, followed by *Acinetobacter* (Figure 1B). The dominant genus in the Tianjin population was *Rickettsiella*, accounting for more than 70%, followed by *Candidatus_Nasuia* and *Pectobacterium* (Figure 1B). When we determined the WDV transmission efficiency of these six populations, all populations were greater than 80% and did not differ significantly among the populations (Figure 1D).

### Differences in the Gut Bacterial Communities in Geographic Populations of *P. alienus*

We used the Chao 1 and ACE indices to evaluate alpha diversity across the different populations. The results showed a saturating number of OTUs by rarefaction curves (Supplementary Figure S3), indicating adequate sampling of 16S rDNA sequences for all the samples. On the basis of the Chao 1 (Supplementary Figure S4A) and ACE index (Supplementary Figure S4B), bacterial diversity in the Linfen population was the highest in all populations, the lowest in the Taiyuan, Baoding and Tianjin population, intermediate in the Tianshui and Hancheng populations, and highest in the Linfen population (Supplementary Figures S4A,B).

**FIGURE 2:**
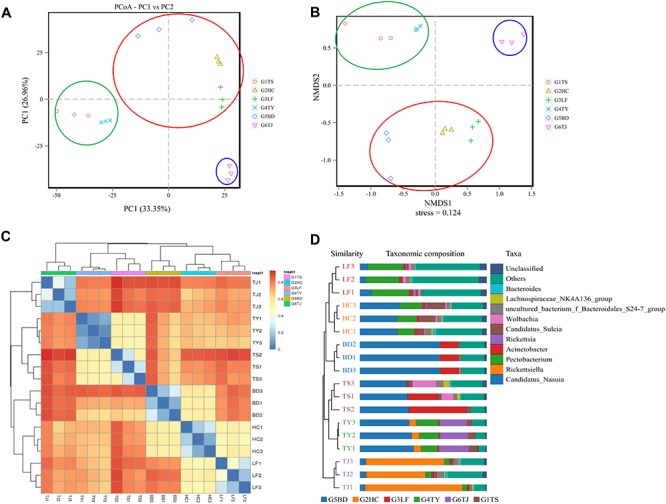
Similarity of bacterial communities of leafhoppers among six locations in China. PCoA analysis **(A)**, NMDS analysis **(B)**, heatmap analysis **(C)**, and UPGMA **(D)**. G1TS: Tianshui; G2HC: Hancheng; G3LF: Linfen; G4TY: Tianjin; G5BD: Baoding; G6TJ: Tianjin.

In an analysis of the beta diversity of the gut bacterial communities based on PCoA (Figure 2A) NMDS (Figure 2B), the results showed a similar tendency in the difference between the Tianjin population (blue circle) and the other five populations. In addition, the Hancheng and Linfen populations (red circle) always formed a tight cluster, as did the Tianshui and Taiyuan populations (green circle), and the Baoding population was near the cluster with Hancheng and Linfen. The heat map (Figure 2C) and UPGMA (Figure 2D) indicated that the distance between different regions was consistent with the above results.

**FIGURE 3:**
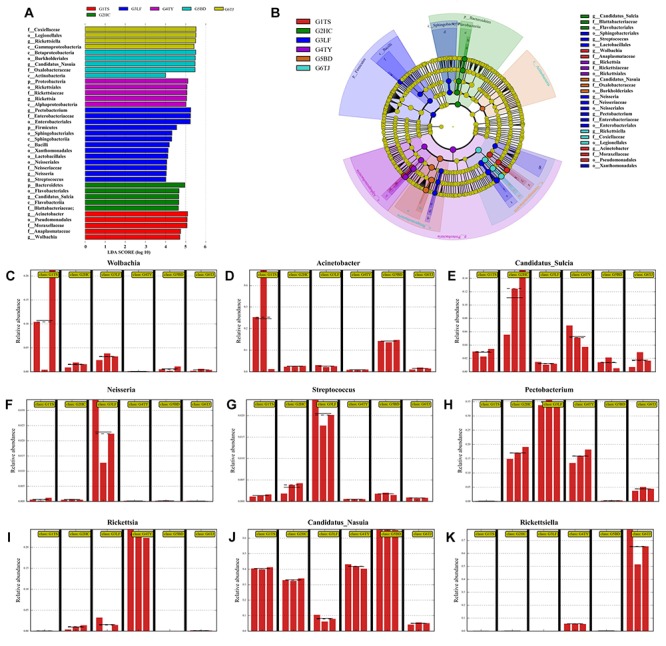
Distinct biomarkers of the bacterial community from different leafhopper populations revealed by linear discriminant analysis (LDA). Multiple regression results are shown for LDA of microbiomes from different populations with LDA > 4. Colors indicate the different populations of the phylogenetic component contributing to group uniqueness **(A)**. Cladogram of the LDA results in panel A. Levels of the cladogram represent, from the inner to outer rings, phylum, class, order, family, and genus. Color codes indicate the six populations, and lower-case letters indicate the taxa that contribute to the uniqueness of the corresponding leafhopper populations **(B)**. **(C–K)** Relative abundance of distinct bacteria in the different populations: *Wolbachia* **(C)**, *Acinetobacter* **(D)**, *Candidatus_Sulcia* **(E)**, *Neisseria* **(F)**, *Streptococcus* **(G)**, *Pectobacterium* **(H)**, *Rickettsia* **(I)**, *Candidatus_Nasuia* **(J)**, *Rickettsiella* **(K)**. G1TS: Tianshui; G2HC: Hancheng; G3LF: Linfen; G4TY: Tianjin; G5BD: Baoding; G6TJ: Tianjin.

In a LDA to determine the bacteria that differed significantly among the six geographic populations, *Wolbachia* (Figure 3A,C) and *Actinobacteria* (Figure 3A,D) accounted significantly for the divergence of the bacterial community in the Tianshui population from the other five populations. *Candidatus_Sulcia* (Figure 3A,E) was an important biomark for the Hancheng population. The genera *Neisseria* (Figure 3A,F), *Streptococcus* (Figure 3A,G) and *Pectobacterium* (Figure 3A,H) distinguished the Linfen population from the other groups. The presence of *Rickettsia* (Figure 3A,I) in the Taiyuan population was a distinct difference from the other five populations. The most distinctive difference in bacterial abundance in the Baoding population was *Candidatus_Nasuia* (Figure 3A,J), whereas *Rickettsiella* differentiated the Tianjin population from the other populations (Figure 3A,K). The OTUs represented in each population are illustrated by phylogenetic levels from phylum to genus in a cladogram (Figure 3B).

### Functional Prediction of the Gut Bacterial Community

In a pathway analysis of the microbes using the Kyoto Encyclopedia of Genes and Genomes (KEGG) database based on their presence within the genomes, bacterial communities in the six natural populations were involved in 43 pathways including amino acid metabolism, carbohydrate metabolism, cofactor and vitamin metabolism (Supplementary Figure S5A). However, the relative abundance of these functions varied among the six groups (Supplementary Figures S5B–E). The membrane transport pathway was predicted to be significantly lower in the microbiota of Tianjin population than in that of the Hancheng, Linfen, Taiyuan and Baoding populations (Supplementary Figures S5B–E). Fewer microbes were involved in signal transduction in Tianjin populations than Hancheng, Taiyuan and Baoding populations (Supplementary Figures S5B,D,E). Significantly more microbes with energy metabolism function were present in Taiyuan, than in Tianjin, followed by the Hancheng and Baoding populations (Supplementary Figures S5B,D,E).

### Composition and Abundance of the Gut Bacterial Community Change Dynamically During WDV Acquisition

**FIGURE 4:**
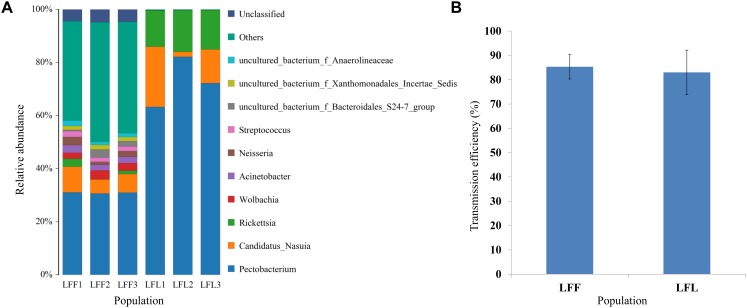
Comparison of gut bacterial community composition **(A)** and virus transmission efficiencies **(B)** between field populations (LFF) from China and laboratory-maintained leafhoppers (LFL). Two nonviruliferous populations (NV) were originally collected from Linfen in 2010. Only the top 10 most abundant are shown.

In a comparison of the gut microbe composition in the nonviruliferous laboratory reared leafhoppers (derive from Linfen) to that in the field-collected leafhoppers from Linfen, the laboratory-maintained leafhoppers had fewer microbes and lower diversity (Figure 4A). However, the virus transmission efficiencies do not have significant difference (Figure 4B).

**FIGURE 5:**
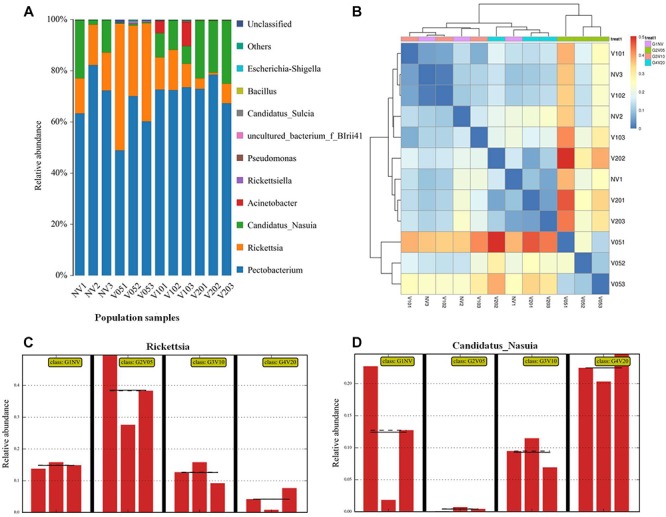
Composition and abundance of the gut bacterial community during the WDV acquisition period. Composition of gut bacterial community at the genus level **(A)**. Only the top 10 most abundant are shown. Comparison of similarities of leafhopper gut bacterial communities at various times during the WDV acquisition period based on Heat map analysis **(B)**. Relative abundance of *Rickettsia* **(C)** and *Candidatus_Nasuia* **(D)** during WDV acquisition. NV: nonviruliferous leafhopper; V05: viruliferous leafhopper after 5 d AAP; V10: viruliferous leafhopper after 10 d AAP; V20: viruliferous leafhopper after 20 d AAP.

We further used the laboratory reared leafhoppers to study gut bacterial community composition in various times (acquisition access period [AAP] of 0, 5, 10, and 20 d) during WDV acquisition. At all four sampling times of laboratory-maintained leafhoppers, *Pectobacterium*, *Rickettsia* and *Candidatus_Nasuia* accounted for over 90% of the microbes, but *Pectobacterium* has similar relative abundance, whereas *Rickettsia* and *Candidatus_Nasuia* changed in relative abundance with increasing duration of access to the WDV-infected plants (Figure 5A). After 5 d AAP, the relative abundance of *Rickettsia* was upregulated and accounted for more than 40% of the bacterial microbiome compared to 15% in the controls. By 10 d AAP, the percentage of *Rickettsia* fell to about 15%, and by 20 d AAP, the *Rickettsia* had decreased to less than 10% (Figure 5C). Conversely, the proportion of *Candidatus_Nasuia* among the total bacteria had severely dropped in the leafhopper by 5 d AAP compared to the control, but had begun rising by 10 d AAP and continued to rise in abundance through 20 d AAP, indicating that WDV impacted the composition of the gut bacterial microbiota in a dynamic and reversible fashion (Figure 5D). Several minor species such as *Acinetobacter*, *Pseudomonas*, *Candidatus_Sulcia* and *Wolbachia* were also identified, but they did not change in relative abundance, i.e., consistent among different samples. The relative abundances of all genera are listed in Supplementary Table S2. Beta diversity was used to evaluate the degree of similarity of bacterial microbiota associated with different times. The results based on a heat map (Figure 5B) showed that the composition of the gut bacterial microbiota from healthy leafhopper, viruliferous leafhoppers after 10 d AAP and 20 d AAP clustered together. In addition, viruliferous leafhoppers after 5 d AAP did not cluster together with the other three groups (healthy leafhopper, viruliferous leafhoppers after 10 d AAP and 20 d AAP). These results further confirm that the composition and abundance of the gut bacterial community recovered during WDV acquisition.

## Discussion

High-throughput sequencing of insect gut microbial community has unveiled vital microbial functions such as cellulose degradation and essential amino acids synthesis that complement metabolic pathways of the host (Lee et al., 2017; Smith et al., 2017). However, there were few studies on the gut bacterial community of sap-sucking insects which transmit various viruses (Jupatanakul et al., 2014). Thus, we focused here on the gut bacterial community of *P. alienus*, which widely distributes in Europe, Asia and Africa and also transmits several cereal-infecting viruses including WDV. Previous studies on microbes in leafhoppers from subfamily *Cicadellidae* using traditional methods have identified members of the bacteria *Sulcia, Sodalis-like* and *Pectobacterium* as resident in the green leafhopper *Cicadella viridis* (Michalik et al., 2014) and *Rickettsia* in the green rice leafhopper *Nephotettix cincticep* (Noda et al., 2012). Recently, Kobialka et al. (2018) examined symbiotic microbes associated with 13 species of *Deltocephalinae* using traditional methods and found several highly abundant microorganisms. Here, we found 22 phyla and 249 genera bacteria in the gut of *P. alienus* from six regions in China using high-throughput sequencing. The bacteria composition and abundance varied among the geographical locations.

The gut bacterial community of *P. alienus* also clustered into different phylogenetic groups depending on the geographical location, suggesting that environment influences the diversity of the gut bacterial communities in the leafhoppers. Tianjin is a coastal city on the Bohai Sea with a typical semi-humid monsoon climate and less temperature and humidity changes than in inland areas. In contrast, the other five regions are far from the sea and have a continental monsoon climate. This means that climate may contribute to the differences in the gut bacterial communities between Tianjin and the other five populations, because the Tianjin population is located on an independent branch in the PCoA and UPGMA analysis. According to climatic data from the China National Meteorological Information Center,^4^ average annual rainfall was about 400–500 mm in the other five regions, but more than 650 mm in Tianjin. Further, the food sources of *P. alienus* in these five regions are similar, i.e., cereal crops and gramineous weeds. Mean annual temperature in Tianshui and Taiyuan is around 10°C, without any extreme high or low temperature during the year. However, mean annual temperature in Hancheng, Linfen and Baoding is between 12.6 and 13.5°C, with the extreme high around 41°C and low about -20°C, indicating a large annual temperature difference. The difference in temperature may explain the high similarity in the gut microbiota among populations of Hancheng, Linfen and Baoding. Previous studies have also shown that the gut microbiome of the scarab beetle (*Holotrichi*a *parallela*) from 10 locations in China was also determined by environmental heterogeneity (Huang and Zhang, 2013). High temperature would also affect normal growth and survival of green stinkbug (*Nezara viridula*) by suppressing obligate gut bacterial symbionts (Kikuchi et al., 2016). Therefore, climate had a greater impact than geographic latitude on the composition and diversity of the gut bacterial community of *P. alienus*. Large differences in annual temperature and humidity could alter the bacterial composition and diversity, and assist insect survival in these local environments.

*Psammotettix alienus* may survive better in different regions through the contribution of various gut bacteria, but whether these bacteria influence virus transmission is not known. Various microbes in insect vector of animal or plant viruses are known to affect viral infection capacity, such as virus entry into host cells. (Moreira et al., 2009; Kliot et al., 2014). In *Aedes albopictus*, the symbiont *Wolbachia* did not affect replication of dengue virus, but it reduced the titre of viruses that entered host cells, thus leading to lower virus transmission efficiency (Mousson et al., 2012). Another symbiont *Chromobacterium* in *A. aegypti* can also reduce mosquito susceptibility to dengue virus infection, resulting in lower virus titre in the mosquito midgut cells (Jupatanakul et al., 2014). Other bacteria such as *Rickettsia* apparently enable *B. tabaci* to acquire more TYLCV from infected plants and promote the virus transmission (Kliot et al., 2014). Because some symbionts can alter virus titre in the insect vector and subsequent transmission efficiency to a new host, therefore we examined WDV transmission efficiency of six geographic *P. alienus* populations. The composition and abundance of bacterial communities differed among the six populations, but WDV transmission efficiency was similar among all populations. Although a much higher level of *Rickettsia* existed in the Taiyuan population than in the others, the virus transmission efficiency showed no significant difference among these populations. Comparing with the field populations, the gut bacterial diversity in laboratory reared leafhoppers decreased obviously, but the WDV transmission efficiency was not affected. Thus, the composition and diversity of the gut bacterial community in *P. alienus* apparently did not affect transmission characteristic as those in other insects.

A previous study showed that infection of the human pathogen *Leishmania infantum* could decrease the bacterial richness in the gut of the vector *Lutzomyia longipalpis* (Kelly et al., 2017). Another study indicated that Marek’s disease virus modifies the core gut microbiome of chickens during the early and late phases of viral replication by enriching several specific genera that might influence inflammation and immunosuppression of T and B cells (Perumbakkam et al., 2014). Interestingly, in the present study, WDV changed the composition and abundance of some gut bacteria which eventually recovered as the AAP lengthened. Relative abundance of *Rickettsia* had increased but *Candidatus_Nasuia* decreased by five AAP, then *Rickettsia* decreased and *Candidatus_Nasuia* increased until the levels resembled those of the nonviruliferous group by 10 AAP, indicating that WDV only impacted some bacteria for a short period. We propose that, unlike the effects of replicating pathogens, a persistent, non-propagative virus might only influence bacterial abundance in the early acquisition phase to enable their entry into the insect vector. Finally, these bacterial changes would progressively recover in the later stage.

In conclusion, resident bacterial community in the gut of *P. alienus* were distributed among 22 phyla and 249 genera; the main genera were *Pectobacterium, Acinetobacter, Candidatus_Nasuia, Rickettsiella, Candidatus_Sulcia, Rickettsia* and *Wolbachia*. The composition and abundance of the bacterial community in *P. alienus* varied among the six geographic regions apparently due to local conditions, whereas the diversity in bacterial functions was similar except for differences in the relative abundance of some functions. However, diversity in the gut bacterial composition did not affect transmission efficiency of the virus as those in other insect vectors. As a persistent, circulative, non-propagative virus, WDV only influenced the abundance of some gut bacteria during early acquisition; the change was dynamic and recovered later in the acquisition period. Our results help to elucidate the complex bacterial communities in leafhopper populations and provide important information for further studies on the complex interactions among the insect vector, microbial symbionts, and the vectored virus and on their coevolution.

## Data Availability

All the data were already available under SRA accession number PRJNA495407.

## Author Contributions

XW designed the experiments and reviewed the manuscript. XW, HW, and WL analyzed the data and wrote the manuscript. HW, WL and JK did preliminary data processing, analysis and manuscript correction. HW, YL and NW processed samples, isolated and sequenced DNA. XW, HW, NW and WL collected samples and edited the manuscript. All authors read and approved the final manuscript.

## Conflict of Interest Statement

The authors declare that the research was conducted in the absence of any commercial or financial relationships that could be construed as a potential conflict of interest.

## Footnotes

1 https://www.arb-silva.de/0

2 https://www.mothur.org/

3 www.arb-silva.de

4 http://data.cma.cn/

## Supplementary Material

FIGURE S1 Scheme of experiment design. **(A)** The composition of gut bacterial community and WDV transmission efficiency in the leafhopper populations collected from six geographic regions. **(B)** Composition and abundance of gut bacterial community in laboratory reared leafhoppers during WDV acquisition. **(C)** Comparison of the gut bacterial community and WDV transmission efficiency between laboratory reared and field collected leafhoppers.

FIGURE S2 Location information of the sampling of *Psammotettix alienus* conducted in this study. TS: Tianshui; HC: Hancheng; LF: Linfen; TY: Tianjin; BD: Baoding; TJ: Tianjin.

FIGURE S3 Rarefaction curves for ‘observed OTUs’ for six field populations. For all gut samples, the number of observed species plateaued as sequence counts increased. G1TS: Tianshui; G2HC: Hancheng; G3LF: Linfen; G4TY: Tianjin; G5BD: Baoding; G6TJ: Tianjin.

FIGURE S4 Diversity indices of the bacterial communities in leafhoppers from the six locations in China. Differences in the Chao 1 index **(A)** and ACE index **(B)** for different geographic locations. G1TS: Tianshui; G2HC: Hancheng; G3LF: Linfen; G4TY: Tianjin; G5BD: Baoding; G6TJ: Tianjin.

FIGURE S5 Comparison of predicted KEGG pathways of gut microbiome from six field leafhopper populations from China. Classification of gene functions **(A)**. Significant difference analysis in relative abundance of metabolic pathways from microbiota functional genes between two populations **(B–E)**. All statistically significant pathways were determined using Fisher test. *p* ≤ 0.01. G1TS: Tianshui; G2HC: Hancheng; G3LF: Linfen; G4TY: Tianjin; G5BD: Baoding; G6TJ: Tianjin.
