# Supplementary figures and images for: Higher Bacterial Diversity of Gut Microbiota in Different Natural Populations of Leafhopper Vector Does Not Influence WDV Transmission
Source: Front Microbiol. 2019 May 29;10:1144. doi: 10.3389/fmicb.2019.01144 (PMC6548887; doi:10.3389/fmicb.2019.01144)

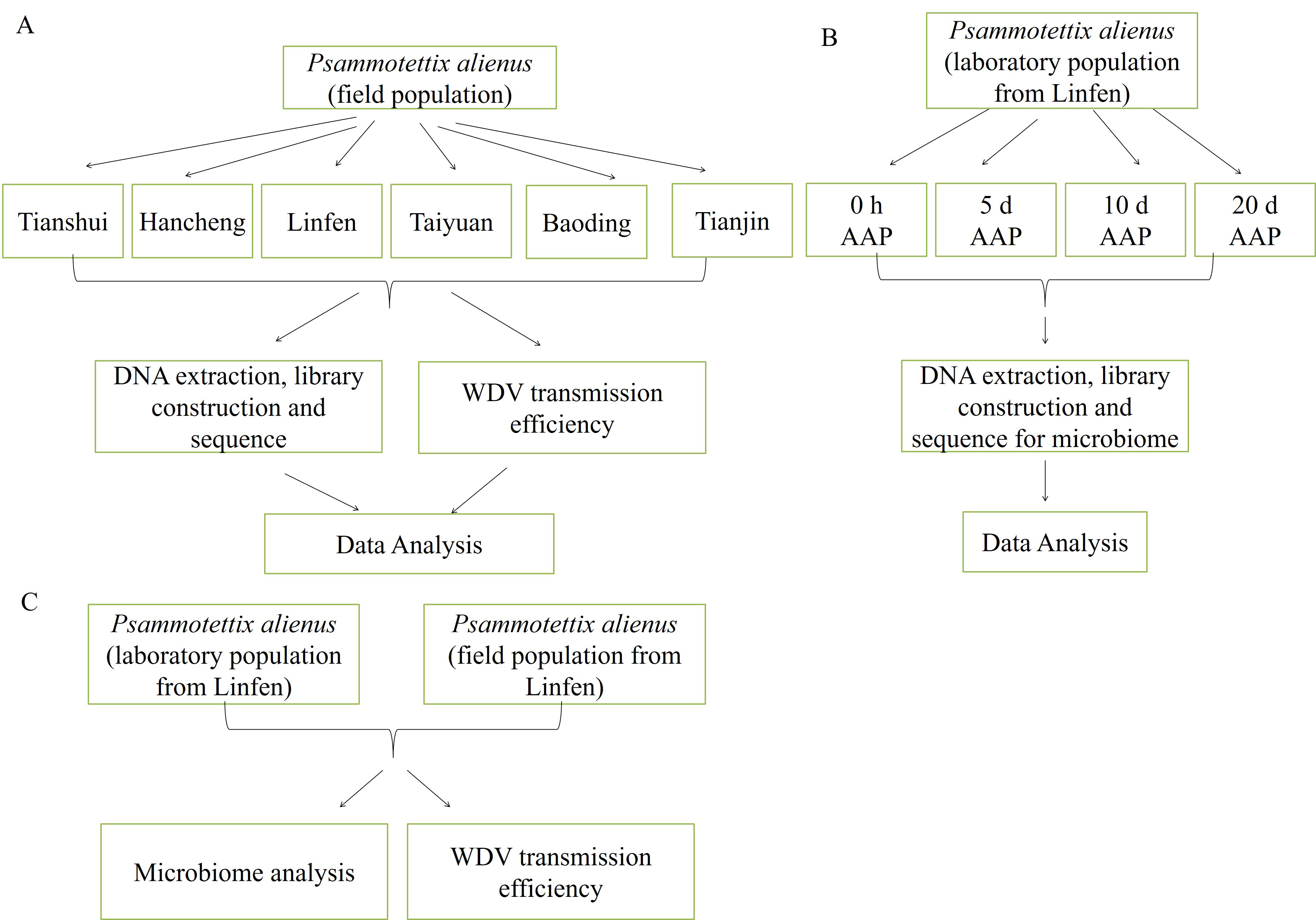

Supplement: FIGURE S1 — Scheme of experiment design. (A) The composition of gut bacterial community and WDV transmission efficiency in the leafhopper populations collected from six geographic regions. (B) Composition and abundance of gut bacterial community in laboratory reared leafhoppers during WDV acquisition. (C) Comparison of the gut bacterial community and WDV transmission efficiency between laboratory reared and field collected leafhoppers. [file Image_1.TIF]

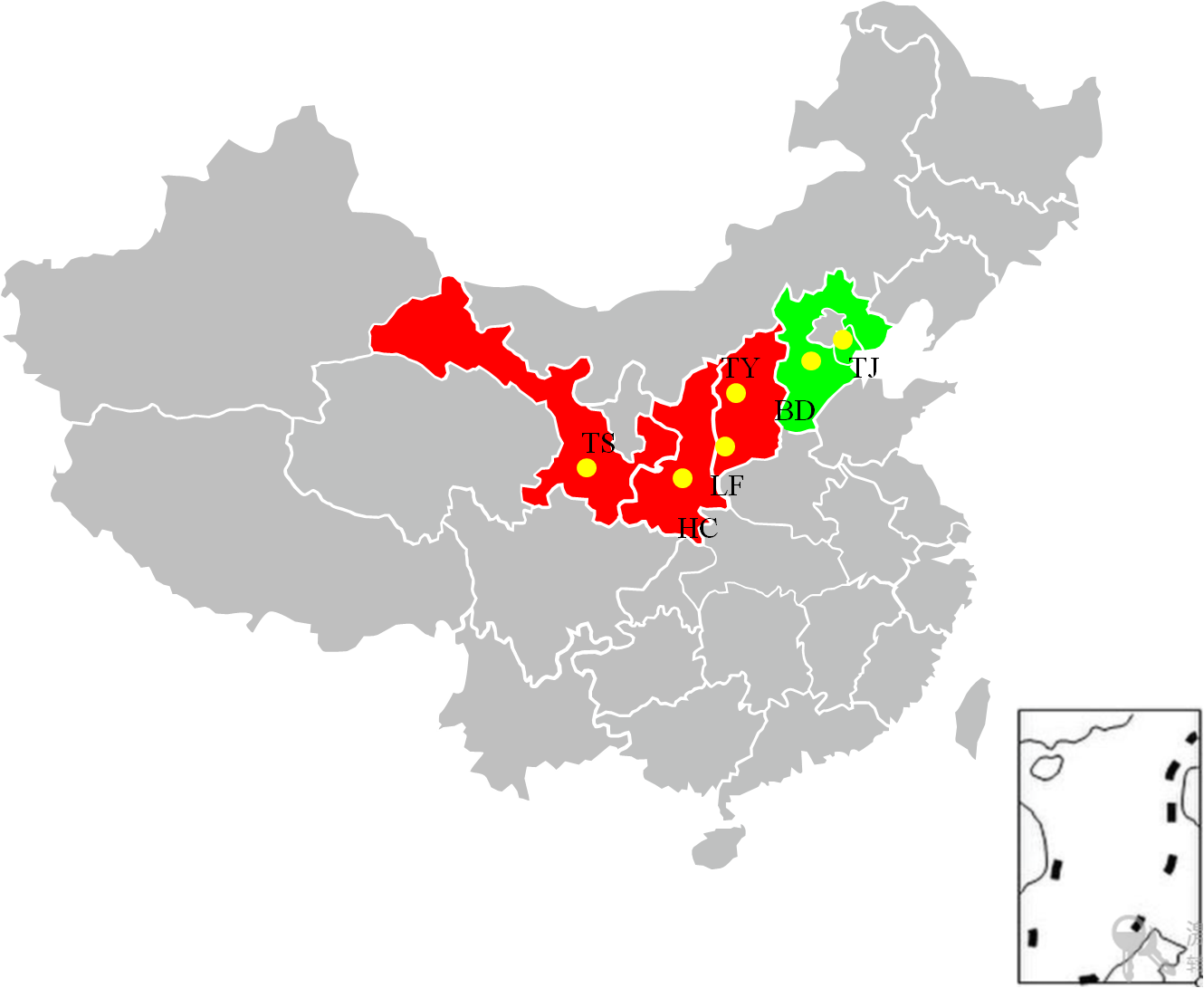

Supplement: FIGURE S2 — Location information of the sampling of Psammotettix alienus conducted in this study. TS: Tianshui; HC: Hancheng; LF: Linfen; TY: Tianjin; BD: Baoding; TJ: Tianjin. [file Image_2.TIF]

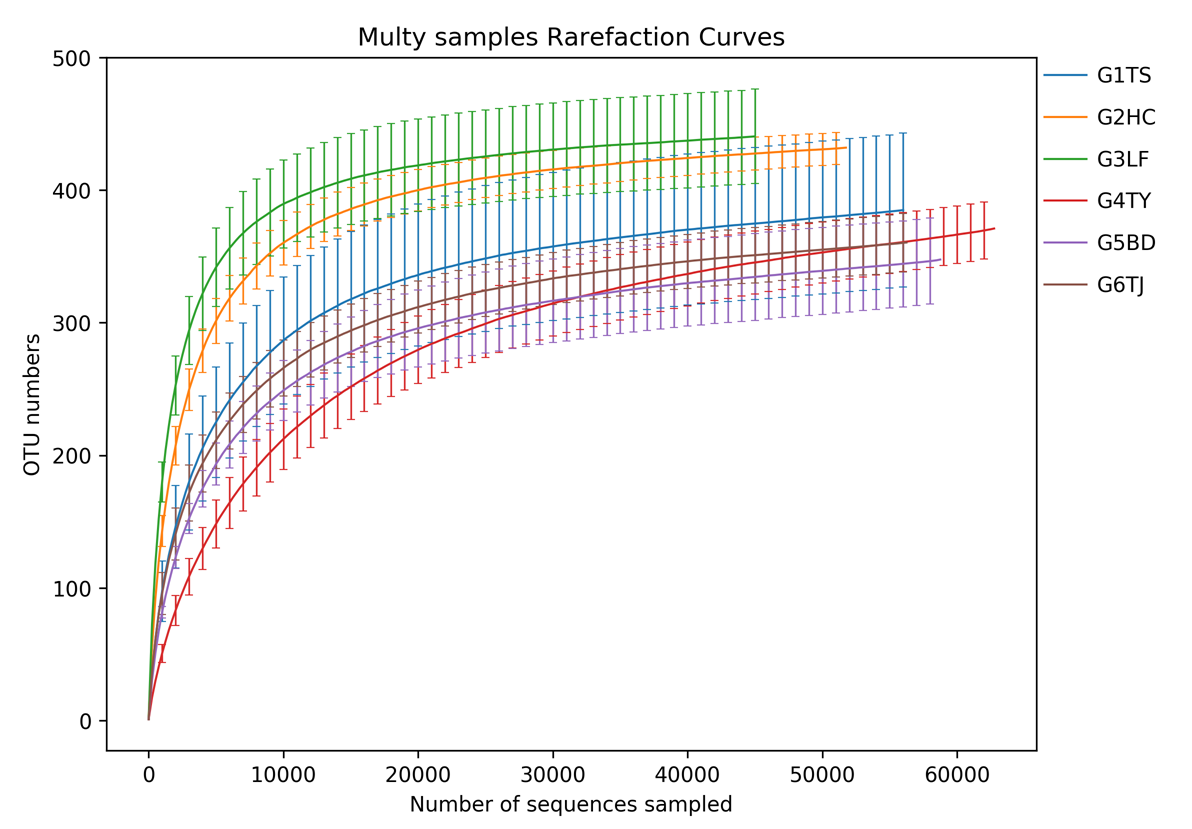

Supplement: FIGURE S3 — Rarefaction curves for ‘observed OTUs’ for six field populations. For all gut samples, the number of observed species plateaued as sequence counts increased. G1TS: Tianshui; G2HC: Hancheng; G3LF: Linfen; G4TY: Tianjin; G5BD: Baoding; G6TJ: Tianjin. [file Image_3.TIF]

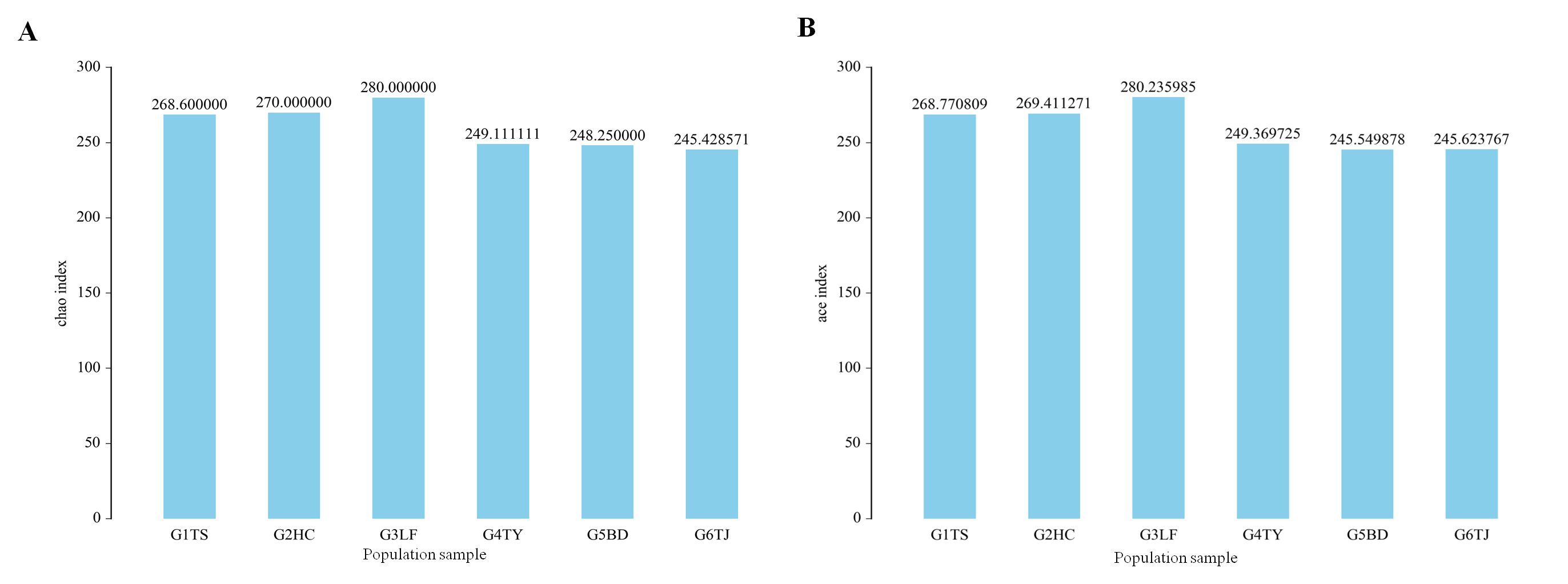

Supplement: FIGURE S4 — Diversity indices of the bacterial communities in leafhoppers from the six locations in China. Differences in the Chao 1 index (A) and ACE index (B) for different geographic locations. G1TS: Tianshui; G2HC: Hancheng; G3LF: Linfen; G4TY: Tianjin; G5BD: Baoding; G6TJ: Tianjin. [file Image_4.TIF]

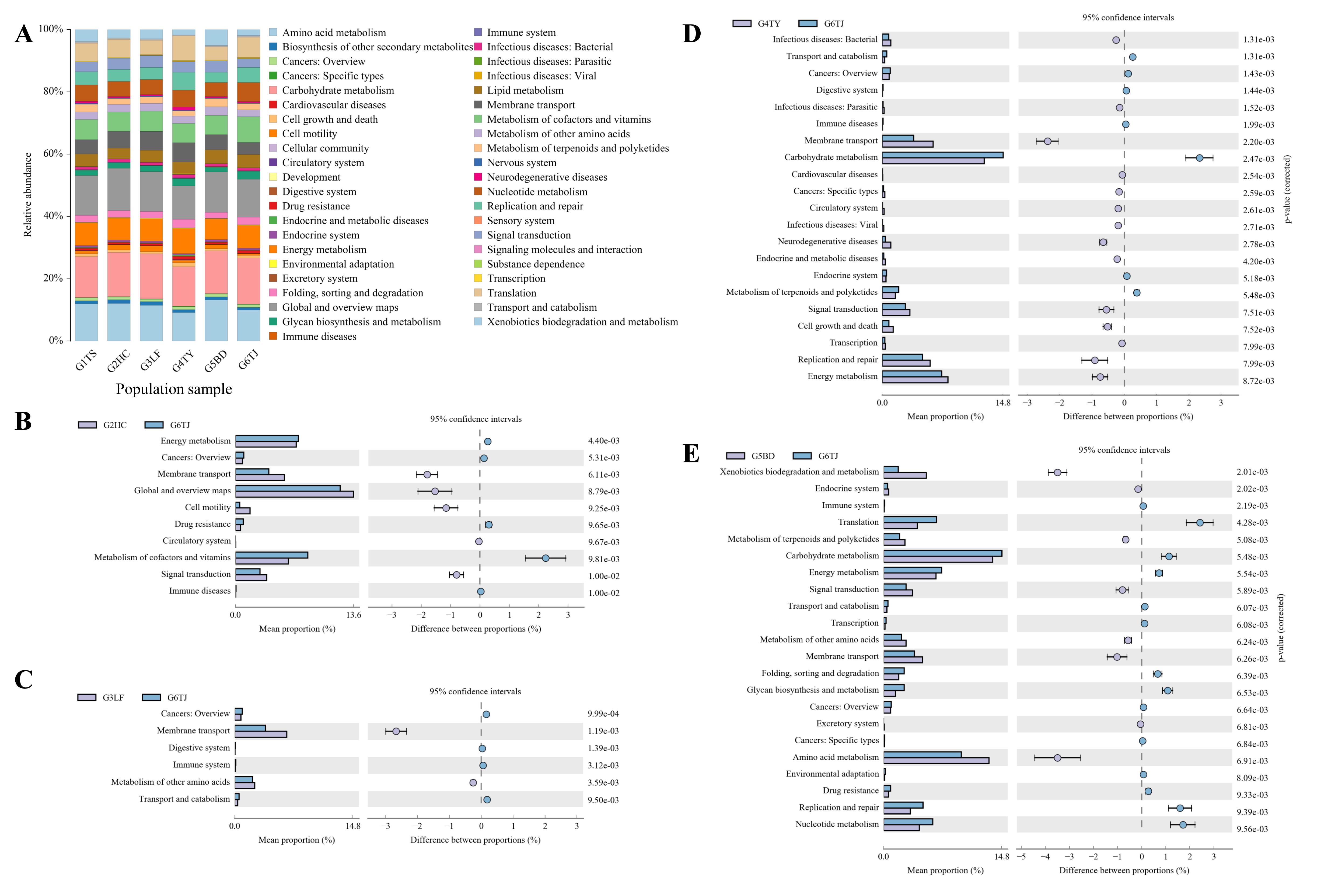

Supplement: FIGURE S5 — Comparison of predicted KEGG pathways of gut microbiome from six field leafhopper populations from China. Classification of gene functions (A). Significant difference analysis in relative abundance of metabolic pathways from microbiota functional genes between two populations (B–E). All statistically significant pathways were determined using Fisher test. p ≤ 0.01. G1TS: Tianshui; G2HC: Hancheng; G3LF: Linfen; G4TY: Tianjin; G5BD: Baoding; G6TJ: Tianjin. [file Image_5.TIF]
